# Linking Genes and Brain Development of Honeybee Workers: A Whole-Transcriptome Approach

**DOI:** 10.1371/journal.pone.0157980

**Published:** 2016-08-04

**Authors:** Christina Vleurinck, Stephan Raub, David Sturgill, Brian Oliver, Martin Beye

**Affiliations:** 1 Institute of Evolutionary Genetics, Heinrich-Heine University, Düsseldorf, Germany; 2 Centre for Information and Media Technology, Heinrich-Heine University, Düsseldorf, Germany; 3 Laboratory of Cellular and Developmental Biology, NIDDK, Bethesda, Maryland, United States of America; University of North Carolina, Greensboro, UNITED STATES

## Abstract

Honeybees live in complex societies whose capabilities far exceed those of the sum of their single members. This social synergism is achieved mainly by the worker bees, which form a female caste. The worker bees display diverse collaborative behaviors and engage in different behavioral tasks, which are controlled by the central nervous system (CNS). The development of the worker brain is determined by the female sex and the worker caste determination signal. Here, we report on genes that are controlled by sex or by caste during differentiation of the worker’s pupal brain. We sequenced and compared transcriptomes from the pupal brains of honeybee workers, queens and drones. We detected 333 genes that are differently expressed and 519 genes that are differentially spliced between the sexes, and 1760 genes that are differentially expressed and 692 genes that are differentially spliced between castes. We further found that 403 genes are differentially regulated by both the sex and caste signals, providing evidence of the integration of both signals through differential gene regulation. In this gene set, we found that the molecular processes of restructuring the cell shape and cell-to-cell signaling are overrepresented. Our approach identified candidate genes that may be involved in brain differentiation that ensures the various social worker behaviors.

## Introduction

Honeybees (*Apis mellifera*) live in complex and structured societies. These societies consist of thousands of usually sterile female workers, a female queen and hundreds of males (drones) [1]. The honeybee workers have a distinct morphology and display social behaviors that are very different from those of queens and drones. Honeybee workers are usually sterile and show altruistic behaviors, including brood rearing, colony defense, and pollen and nectar foraging behavior. All of these behaviors are devoted to maintaining the colony [2–4]. The queens and drones display behaviors that are associated with reproduction, including mating and egg laying by queens. The behaviors of social workers are of special interest because the collaborative and seemingly coordinate behaviors of thousands of workers produce outcomes that far exceed the abilities of a single worker [3, 5, 6]. Workers can collectively thermoregulate a bee colony [7] or exploit food sources. Worker bees utilize sophisticated communication systems (e.g., the waggle dance behavior) in which the quality, distance and direction of a food source is communicated to other workers [2]. Moreover, worker bees exhibit an age-dependent division of labor [3, 8, 9] in which newly emerged bees perform tasks inside the nest, whereas older bees are engaged in foraging behavior. This age-dependent polyethism is, however, not fixed. Honeybee workers flexibly engage in certain tasks depending on the colonies' needs [9, 10]. Even a foraging worker bee reverts physiologically and behaviorally to a nursing bee on demand [11–13]. We suggest that the wiring of the worker brain, which is specified during pupal development, ensures the ability to perform different worker tasks. In *Drosophila melanogaster*, much of the wiring of the adult brain is specified during pupal development by a genetically determined program [14, 15]. We have little understanding of how a developmental program can specify a brain that controls and ensures such sophisticated innate social behaviors as displayed by the honeybee workers. This developmental program of worker honeybees originated in the last 60 to 75 million years [16] from a solitary bee ancestor.

**Fig 1:**
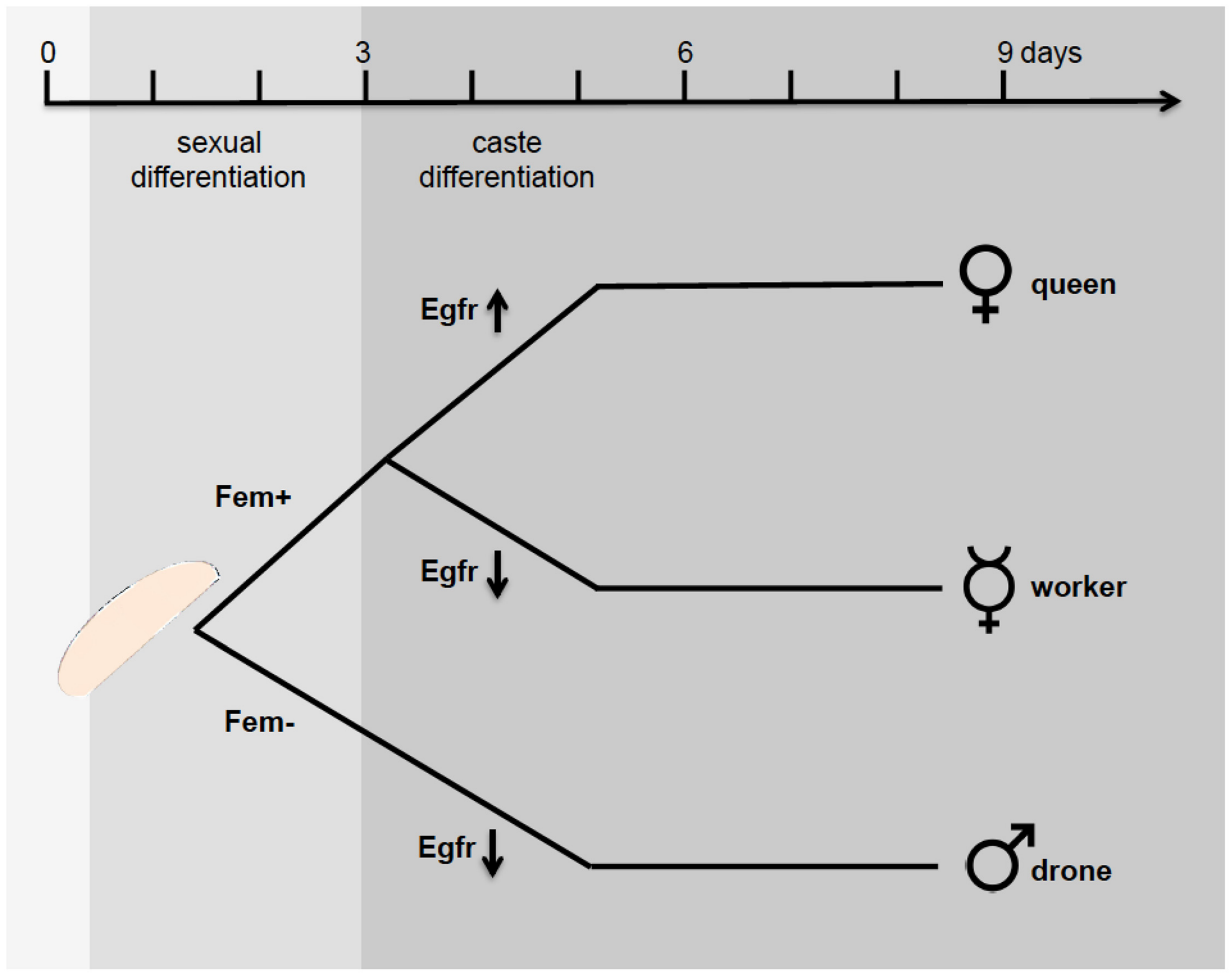
The control of sexual and caste development in honeybees. Sexual differentiation is induced approximately 12 hours after egg laying and is determined by the genotype at the *csd* gene, which directs splicing of the *feminizer* (*fem*) transcripts. The female *fem* transcripts encode Fem proteins, which maintain the female-determined state through a positive feedback loop. The female caste differentiation into workers and queens is determined by the differential feeding of larvae with royal jelly. This feeding differentially regulates the Epidermal growth factor receptor (Egfr).

In the honeybee, the combined action of the sex- and caste-determining pathways specifies worker differentiation, including in the brain. The sex determination pathway regulates the differences between the sexes [17–19], while *Epidermal growth factor receptor* (*Egfr*) signaling regulates differentiation into the different female castes of workers and queens [20]. Femaleness is induced by a heterozygous genotype at the *complementary sex determiner* (*csd*) gene. The heterozygous Csd proteins direct female splicing of *feminizer* (*fem*) transcripts that switches the pathway into the female state [17–19]. The female *fem* transcripts encode the splice regulator Fem, which directs female splicing of transcripts; among these transcripts, the honeybee *doublesex* (*Am-dsx*) gene has been identified [18]. Males develop when the *csd* genotype is hemizygous or homozygous. Those Csd proteins have not the activity to direct female splicing of *fem* transcripts. As a consequence, a male transcript of the *fem* gene, which contains a premature stop codon and encodes no functional Fem protein, is produced by default. In the absence of the Fem protein, male-spliced *Am-dsx* transcripts are produced. Until now, we have had little understanding of the genes that operate further downstream of the *csd* and *fem* genes in executing female development and subsequent worker and queen differentiation. The differentiation into either workers or queens is controlled by differential feeding of the female larvae with royal jelly [20] (Fig 1).

Queen development is induced when female larvae are fed exclusively royal jelly by workers throughout larval development. Worker development results when the amount of royal jelly is restricted in older female larvae. The Royalactin protein (Rol) has been characterized as an active component of the royal jelly, which is produced by the hypopharyngeal glands of workers. Rol induces queen and not worker development through the differential activation of the *Epidermal growth factor receptor* (*Egfr*) pathway (Fig 1) [21]. This altered *Egfr* activity in queens directs an increase in body size and activates ovary development, which are both mediated by the activation of the S6 kinase (S6K) and the mitogen-activated protein kinase (MAPK) signaling pathways [21]. The drones receive a similar shortage of royal jelly feeding in later larval stage [20, 22]. Thus, the drones exhibit the same *Egfr* signaling that is found in workers. So far, we have had no further understanding of which genes further downstream of the *Egfr* pathway regulate the brain differentiation during development of an adult female. These downstream components of the genetically encoded pathway specify the adult brain that orchestrates the very different behaviors displayed by workers and queens.

This study seeks (i) to identify candidate genes downstream of the *fem* and *egfr* pathways that are involved in differentiation of the adult worker brain, and (ii) to detect mechanisms by which the sex- and caste-determining signals are integrated into a genetically encoded program.

Here, we report the candidate genes that are regulated by either the sex or caste determination signals or by the action of both signals during pupal brain differentiation. We compared the transcriptomes of differently expressed and spliced genes in the pupal brains between workers and queens as well between workers and drones using an RNAseq approach. This comparison identified genes that are regulated by the sex- or the caste-determining signals or by both signals in the developing worker brain. We chose the pupal stage (P4 in workers and drones, P3 in queens [23]) because during pupation, the adult brain is specified such that it can orchestrate the worker behaviors and the temporal division of labor [14, 15]. The identified candidate genes provide a resource for the further study of the genetically encoded developmental program of worker brain differentiation during pupal development.

## Materials and Methods

### Bee Sources

Worker and drone pupae (*Apis mellifera* L.) with red pigmented eyes (P4 stage [23]) were collected from sealed brood combs maintained in the beekeeping facility at the Heinrich-Heine University of Düsseldorf (Germany). To produce queen pupae, female eggs were collected using the Jenter egg collecting device (Karl Jenter, Nürtingen, Germany) and were placed into a queenless colony. Worker of the queenless colony reared queens, which were collected in P3 stage 14 days after the eggs were laid. The P4 stage for workers and drones and P3 stage for queens were chosen because there is evidence from gross morphological studies of worker and queen brains that those stages represent similar developmental stages [23], which is externally indicated by the same coloration of the compound eye (the eye color turns red, see [23]).

### RNAseq studies

RNA was isolated using a Trizol based method. The tissues of three brains were homogenized with a pestle in 250 μl Trizol reagent. The mixture was vortexed for 15 seconds and incubated for 5 minutes at room temperature. 50 μl chloroform were added to the samples, which were then vortexed for 30 seconds, incubated for 1 minute at room temperature and vortexed for 2 additional minutes. The samples were then centrifuged for 15 minutes at 4°C and 13,000 rpm. The upper phase was combined with an equal volume of 70% ethanol. The mixture was vortexed and transferred to a spin column (RNeasy MinElute Cleanup Kit, Qiagen, Hilden, Germany). The following washing and elution steps were all performed using the RNeasy MinElute Cleanup Kit and the RNeasy Mini Kit (Quiagen, Hilden, Germany) by following the procedure described in the respective manuals from the provider. We added 350 μl RW1 buffer to the filter cartridge, which was followed by 10 μl RNase-free DNase and 70 μl RDD buffer and incubated for 15 minutes. The washing was performed using 350 μl RW1 buffer, 500 μl RPE buffer and finally 500 μl 80% ethanol. We eluted the RNA using 12 μl RNase free water.

We found no detectable amounts of degraded 18S and 28S RNA using a 2100 Bioanalyzer instrument (Agilent Technologies, Santa Clara, CA, USA), which indicated the isolation of high quality RNA. RNA was prepared for sequencing following the TruSeq RNA Sample Preparation Guide (v2, Illumina, San Diego, CA, USA). Single-read sequencing was performed with the Illumina HiSeq 2000 system (2500 for the queen samples; Illumina, San Diego, CA, USA). The quality of the resulting 100 base long reads was measured using the Phred quality score that was calculated with the CLCbio Genomics Workbench software. We achieved an average Phred score of 34 for all samples, which indicated a base call accuracy of 99.95% and a probability of an incorrect base call of 4x10^-4^. We have provided the data in the Gene Expression Omnibus (GEO) under accession number GSE45408.

For our RNAseq analyses, we aligned the sequences to the NCBI honeybee reference genome (Assembly v.4.5: ftp://ftp.ncbi.nih.gov/genomes/Apis_mellifera/Assembled_chromosomes/seq/) and the NCBI transcript models of the annotated genes (ftp://ftp.ncbi.nih.gov/genomes/Apis_mellifera/GFF/ref_Amel_4.5_top_level.gff3.gz).

We concatenated the chromosome assemblies of the reference genome for linkage groups 1–16 and excluded unplaced sequences and the mitochondrial genome. Minor edits were made to the annotation file due to non-unique identifiers for tRNAs by deleting one of the duplicated identifiers.

We performed a bioinformatics analysis executing all commands with the default settings unless otherwise noted. Read mapping was performed using Tophat (v2.0.8) [24], Bowtie2 (v2.1.0) [25] and Samtools (v0.1.18) [26] accepting only the uniquely mapped reads (option–g 1 in the *tophat* command line). We created a transcript model *de novo* by assembling the mapped reads using the Cufflinks package (v2.0.2) [24]. To annotate the transcription starts, we used the *cuffcompare* command in the Cufflinks package [24]. We added the option–CG to the *cuffcompare* command so that the command accepted a GFF3 file format as the transcript model instead of the standard GTF file format. We ran Cuffdiff (part of the Cufflinks program package) to identify the significant differentially expressed genes (DEGs). We added the option -N to the *cuffdiff* command to indicate 'Upper Quantile Normalization'. This option normalizes for sequencing depth using the upper quartile counts instead of the total read counts.

We used the Spanki software program (v0.4.0)[27] to detect significant differentially regulated splice junctions in our mapped reads. The number of reads mapped to known genes depends on the accuracy and completeness of the transcript model. Because the annotated honeybee transcript model provided by NCBI is not yet complete (especially for genes expressed in queens or drones), we relied on the detection of single differentially spliced junctions instead of identifying differentially spliced transcripts [27].

The identified DEGs and differentially spliced junctions were further managed and visualized using R software and the R package CummeRbund [24], which enabled the further sorting of genes according to their p-values or splicing criteria. The multiple-testing corrected q-values were set to 0.001 for DEGs and differentially spliced junctions. We further decreased the list of splice junctions by setting the criteria as follows: anchor ≥ 8, hamming 3 ≥ 3, hamming 5 ≥ 3, entropy ≥ 2 and dinucleotide = GT-AG (see [27]. We identified the gene name or LOC number for each differentially spliced junction using the Integrative Genome Viewer (IGV [28]) by prompting the nucleotide position of the junction and assigning the gene name given in the transcript annotation. This procedure resulted in the assignment of differently spliced genes in this study even though some single genes displayed more than one differentially spliced junction.

To obtain further information on the DEGs and DSGs, we used the information provided in the NCBI database to annotate the molecular gene functions and their functional classes (protein coding gene, ncRNA (noncoding RNA) or miscRNA (small miscellaneous ncRNA)).

### Gene ontology analysis

The encoded amino acid sequences of differentially expressed or spliced honey bee transcripts were obtained from NCBI and blasted against the *Drosophila melanogaster* protein database using the Blast2GO software [29]. The obtained orthologs were converted into GO annotations using DAVID Bioinformatics Resources (v 6.7; https://david.ncifcrf.gov/) [30]. The GO annotations were analyzed for enriched GO terms using the REVIGO software [31].

## Results

The adult honeybee worker brain is specified during pupal development by the combined action of the sex-determining and caste-determining pathways. To identify genes that are controlled by the sex- and caste-determination signals and that may execute the worker-related brain differentiation, we deep-sequenced the transcriptomes (RNAseq) derived from the developing pupal brains. We compared the transcript strengths and alternative splicing of transcripts controlled by the sex signal (worker/male comparison) and by the caste signal (worker/queen comparison). The worker/male comparison identified the sexual signal-derived controls resulting from the similar feeding of Rol protein to male and worker larvae. The worker/queen comparison identified the caste signal-derived gene regulations under the control of the female pathway. The differentially expressed or spliced transcripts identified in both comparisons indicated genes regulated by the combined action of the sex- and caste-determination pathways and, hence, displayed the integration of both signals.

We examined three biological replicates for each condition (the workers, the queens and the males). Each replicate consisted of three brains derived from the pupal developmental stage during which the eye color began to turn red (P4 in workers and drones, P3 in queens) [23]. On average, 187 million 100 bp reads were obtained per replicate using the Illumina HiSeq2000 or 2500 systems (S1 Table in S1 File). We mapped an average of 81% of our sequence reads to the honeybee genome (S1 Table in S1 File), which is a result similar to those obtained in previous mapping RNAseq approaches in the honeybee [32]. We generated transcripts by assembling the mapped sequence reads using the software program Cufflinks [33]. We estimated the FPKM (**F**ragments **P**er **K**ilobase Of Exon Per **M**illion Fragments Mapped) values as a measure of the expression strength for each gene using the software program Cuffdiff. This allowed us to detect the differentially expressed genes (DEGs; Table 1, S2 Table in S1 File) between conditions. We analyzed the splice junctions of our mapped reads using the software program Spanki [27] and identified the significant differentially spliced genes (DSGs; Table 1, S3 Table in S1 File).

**Table 1:** Differentially expressed and spliced genes in the brains of workers compared to males and in workers compared to queens.

		Comparison	
	Category of genes	Workers Drones	Workers Queens
Differentially expressed genes (DEGs)*	protein coding	282	1623
	ncRNA	12	43
	miscRNA	2	13
	no information	37	81
	total	333	1760
Differentially spliced genes (DSGs)*	protein coding	447	627
	ncRNA	18	15
	miscRNA	4	6
	no information	50	44
	total	519	692

* cut-off value for each gene, *p* < 0.001.

We first studied the correlations of the FPKM values for each gene between biological replicates (S4 Table in S1 File) and each condition (S5 Table in S1 File). We found that these values and approximations of expression strength were highly correlated between biological replicates (90 to 99%, with an average of 96%), but differed substantially between conditions (58% to 94%, with an average of 74%). These results show that the variation between biological replicates are low compared to those between conditions, suggesting that the FPKM values were subject to condition-specific effects.

**Fig 2:**
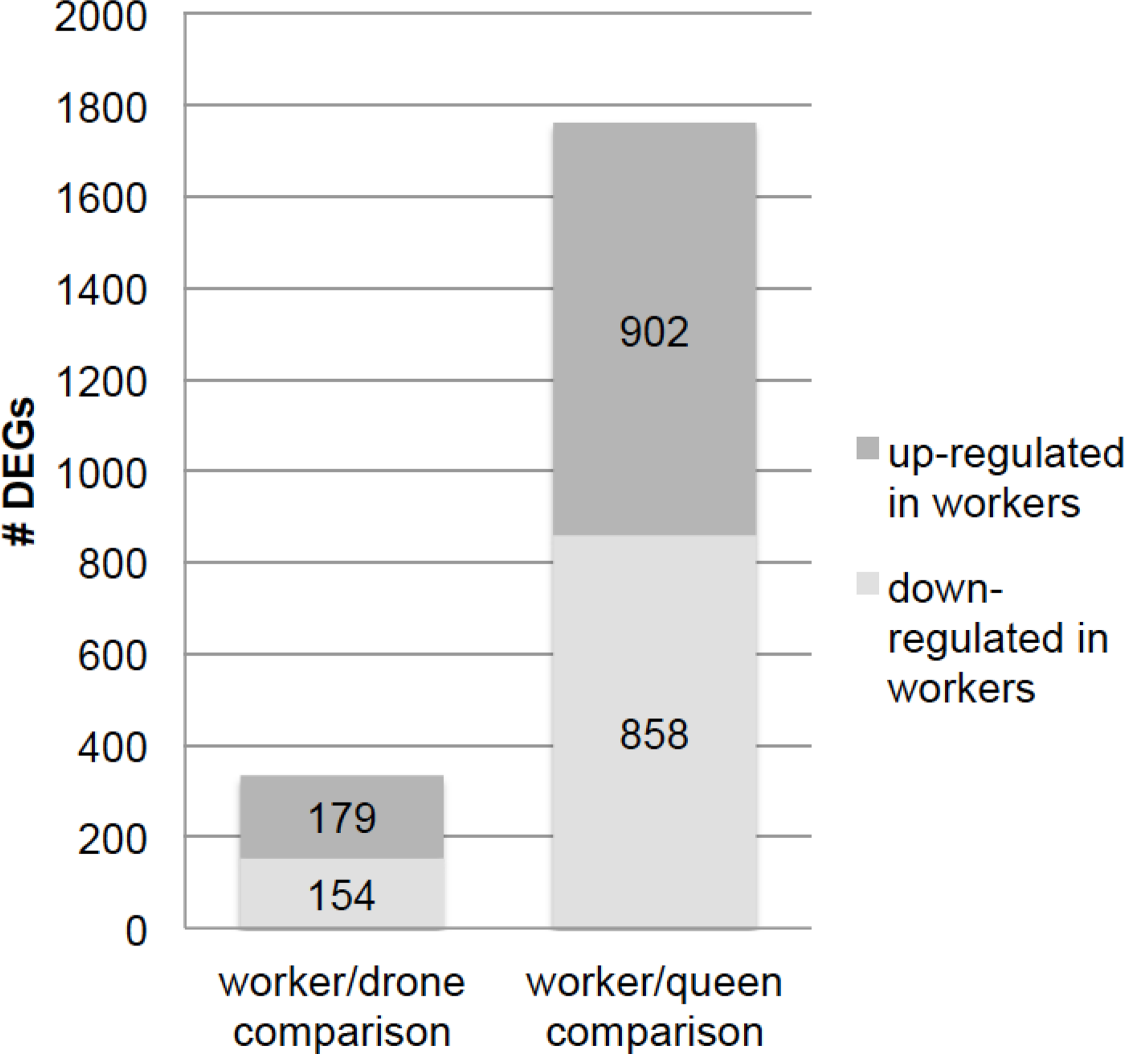
The number of DEGs that were up- and down-regulated in the brains.

We detected 333 genes that showed differences in expression strength between workers and males (Table 1). Overall, 179 of these DEGs were up-regulated, and 154 were down-regulated in workers (Fig 2). Further, 282 of these genes encode proteins, 12 genes transcribe non-coding RNAs (ncRNAs) and 2 express miscellaneous RNAs (miscRNA). We detected 519 genes with differentially spliced transcripts between workers and males (Table 1). Of these, 447 encode proteins, including those that have been previously studied for sex-specific splicing, such as the *fem* and the *Am*-*doublesex* (*Am*-*dsx*) genes [18]. Eighteen differentially spliced transcripts were non-coding RNAs, and 4 were miscellaneous RNAs. We found more genes with differential splicing than with differential expression in the worker/drone comparison (Table 1), suggesting that the sexual signal mostly affects the alternative splicing of transcripts.

We annotated the differentially expressed and spliced protein encoding transcripts with the Gene Ontology (GO) categories cellular components and molecular processes. We functionally annotated the differentially expressed and spliced protein encoding transcripts by comparing the proteins to their homologs in *Drosophila melanogaster* and converting those annotations to Gene Ontology (GO) terms. We found that in the cellular component analysis, genes associated with intrinsic or integral components of the cell membrane (GO:0005576, GO:0031224, GO:0016021, GO:0005886) were overrepresented in the protein encoding genes (282 DEGs and 447 DSGs) (S6 and S8 Tables in S1 File). The molecular processes of cation and ion binding (GO:0043167, GO:0043169) and DNA and RNA binding (GO:0003700, GO:0017076, GO:0032553, GO:0001882, GO:0032555, GO:0001883, GO:0030554, GO:0032559) were also overrepresented (S7 and S9 Tables in S1 File).

For the worker/queen comparison, we found 1760 differentially expressed transcripts (Table 1, S2 Table in S1 File). Similar numbers of DEGs, 902 were up-regulated, and 858 were and down-regulated (Fig 2). In total, 1623 genes were found to transcribe mRNAs encoding proteins, 43 genes to transcribe non-coding RNAs, and 13 genes to transcribe miscellaneous RNAs (Table 1). We detected 692 DSGs in worker and queen brains (S3 Table in S1 File). Of them, 627 encode proteins, 15 genes transcribe non-coding RNAs, and 6 encode miscellaneous RNAs (Table 1). Evaluating the DEGs and DSGs in the worker/queen comparison demonstrated that the caste signal predominantly affected gene expression. The molecular processes of phosphorus metabolic processes (GO:0006793, GO:0006796), cell surface receptor signaling (GO:0007166), RNA metabolism (GO:0051252), DNA-templated transcription (GO:0006351) and nucleotide binding (GO:0000166) were overrepresented in the GO analysis (S10-S13 Tables in S1 File).

**Fig 3:**
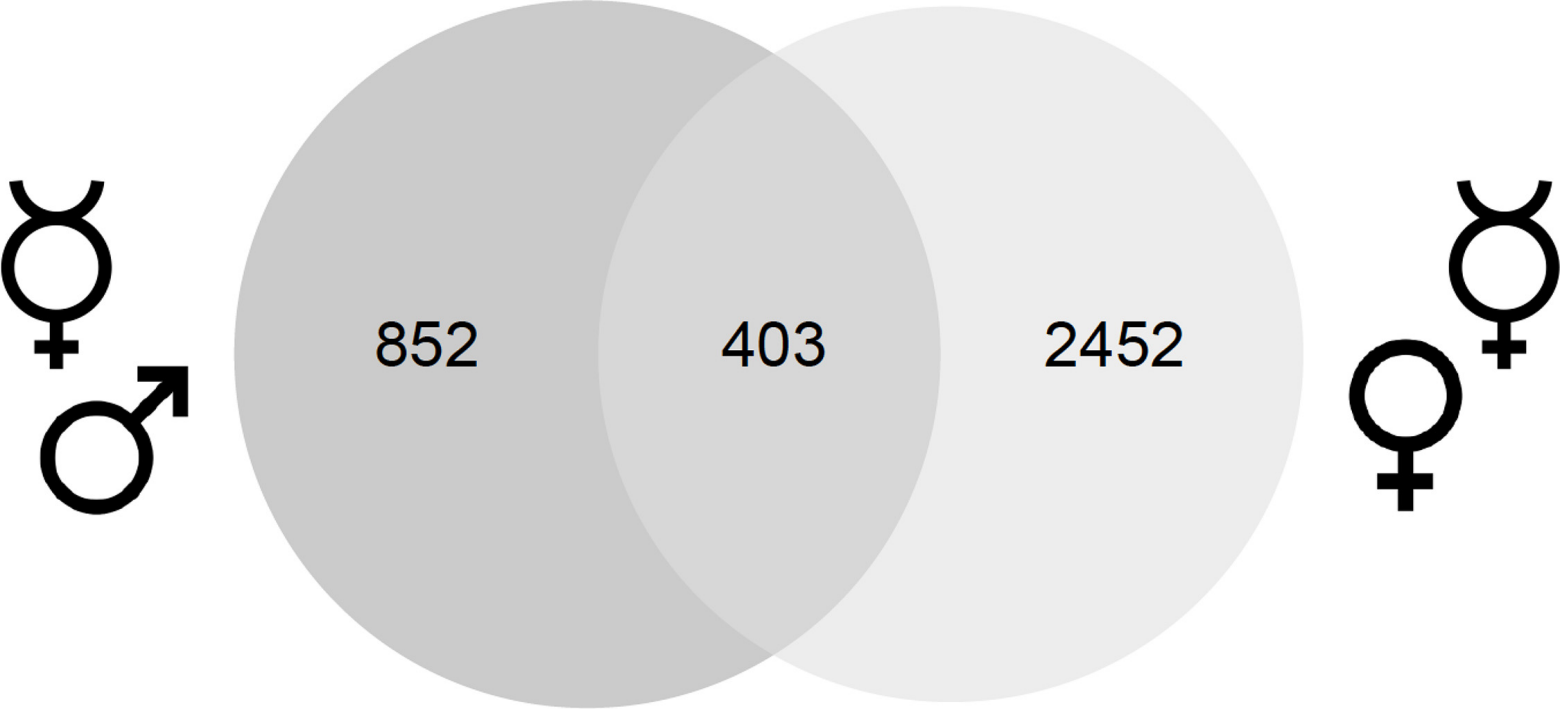
Venn diagram of the number of differentially spliced and expressed genes in the worker/male and worker/queen comparisons.

To detect genes that are controlled by the combined action of the caste and the sex determination pathways, we selected the genes that were differentially regulated in both the worker/male and worker/queen comparisons. Because those genes are regulated by both the sex- and the caste-determining signal, they can integrate the information of both pathways. We found that 204 genes were co-regulated by the sexual and caste signals at the level of gene expression, and 143 genes were co-regulated at the level of transcript splicing (S1 Fig in S1 File). We found that 403 genes were co-regulated by the sexual and caste signals irrespective of their mode of differential regulation (splicing or expression) (Fig 3, S1 Supporting Information in S1 File). 359 of the 403 genes encoded proteins, 15 genes encoded non-coding RNAs, and 5 encoded miscellaneous RNAs. The GO analysis of the 359 protein encoding genes showed that proteins involved in restructuring the cell shape (GO:0005865, GO:0015629, GO:0005523) and in cell-to-cell signaling (GO:0005886, GO:0001633, GO:0005509) were overrepresented (S14 and S15 Tables in S1 File).

The number of genes that are jointly regulated by the two signals was highly overrepresented compared to the number of co-regulated genes expected by chance. The probability of obtaining the observed result of 403 co-regulated genes (Fig 3) is *p* < 0.001. The *p*-value was obtained by applying the hypergeometrical distribution of the 15,791 genes that were transcribed in the brain in our study, the 121 genes that were expected to be co-regulated by chance, and the fraction of genes regulated by the sexual or caste signals. These estimates suggested that the worker bees employ a large set of genes to possibly integrate the sexual and caste information.

## Discussion

We identified sets of differentially expressed and differentially spliced genes (DEGs and DSGs, respectively) that were controlled by the sexual signal, the caste signal, or both signals in the developing brains of pupal worker bees. This approach provides a method for detecting candidate genes involved in the specification of the worker, queen and drone brain to ensure distinct social behaviors later on in adult live. The female signal together with a worker caste signal determines a worker brain that can orchestrate the sophisticated social behaviors that are essential in forming a complex society.

We found that under the control of the sexual signal, genes were more often differentially spliced (3% of the transcribed genes in the brain) than differentially expressed (2% of the transcribed genes), suggesting that sex-dependent splicing plays a major role in executing the sexual signal in the brain. Our gene set included the splice regulator *fem* and the transcription factor *Am-dsx*, which have both been previously identified [18]. Genes that can bind to DNA and RNA were overrepresented in our GO analysis. This result suggests that the action of the sex- and caste-determining pathways are often executed via genes that regulate transcription or post-transcription. In a survey conducted in the brain of the fruit fly (*Drosophila melanogaster*), 18 differentially spliced transcripts, including the *Dm-doublsex* (*dsx)* gene, were identified between the sexes [27]. Disrupting *Dm*-*dsx* neuronal function in *D*. *melanogaster* had profound effects on male sexual behavior and female reproductive behaviors [34] that are associated with the sexual dimorphism of the neuronal wiring.

We suggest that some of our DEGs and DSGs may have similar roles in specifying the sophisticated social behaviors in honeybees, which require, in the case of the worker, both a sexual and a caste signal. Our study demonstrates that the caste regulatory differences in female brains during pupal differentiation, during which the adult brain is specified, are primarily achieved by expression differences (11%). Only 4% of the genes showed differential splicing. The GO analyses indicated that receptor-based signaling and gene regulation via transcription were overrepresented; these molecular processes play an important role in the neuronal wiring and ensure the development of complex behaviors [35]. We found an equal amount of DEGs that were upregulated in queens (49%) and workers (51%). In contrast, Chen et al. [36] found that in the L5 larvae stage, 72% (3467 genes) of the DEGs were up-regulated in queens and 28% (1320) were up-regulated in workers. We suggest that the substantial upregulation of transcription in the larvae may be associated with the fast growth and shorter developmental time of queens during larval development, which is not required during adult brain formation.

We detected 403 genes that were co-regulated by the sexual and caste signal. These genes may play a critical role in worker specific differentiation, as they are regulated by the female and the worker-like caste pathways [21] and can integrate the information from both pathways. Thus, these 403 genes may play a critical role in the developmental specification of the adult worker brain. The GO analysis suggested that the integration of the sexual and caste information is realized through cell signaling processes and cell shape changes in the neuronal substrate. The identified genes (S1 Supporting Information in S1 File) provide a powerful avenue to further study the processes specifying a worker brain to ensure social behaviors and organization. These candidate genes can be further studied using the genetic manipulation method that we recently introduced for honeybees [37].

## Supporting Information

S1 File S1 Figure: Venn diagram of the number of differentially expressed (DEGs) and differentially spliced (DSGs) genes in the worker/male and worker/queen comparisons. S1 Table: Numbers of reads that were sequenced and the numbers and proportions of reads that were mapped to the honeybee genome. S2 Table: Number of differentially expressed genes in the brain between the conditions. S3 Table: Number of differentially spliced genes (DSGs) between the conditions. S4 Table: Correlation coefficient of the FPKM values between the conditions and replicates. S5 Table: The average correlation coefficient of the FPKM values between the different conditions. S6 Table: Cellular Component Gene Ontology (GO) of differentially expressed genes between the pupal brains of worker bees and drones. S7 Table: Molecular Function Gene Ontology (GO) of differentially expressed genes between the pupal brains of worker bees and drones. S8 Table: Cellular Component Gene Ontology (GO) of genes that show significant differential use of splice junctions between the pupal brains of worker bees and drones. S9 Table: Molecular Function Gene Ontology (GO) of genes that show significant differential use of splice junctions between the pupal brains of worker bees and drones. S10 Table: Cellular Component Gene Ontology (GO) of significant differentially expressed genes between the pupal brains of worker bees and queens. S11 Table: Molecular Function Gene Ontology (GO) of significant differentially expressed genes between the pupal brains of worker bees and queens. S12 Table: Cellular Component Gene Ontology (GO) of genes that show significant differential use of splice junctions between the pupal brains of worker bees and queens. S13 Table: Molecular Function Gene Ontology (GO) of genes that show significant differential use of splice junctions between the pupal brains of worker bees and queens. S14 Table: Cellular Component Gene Ontology (GO) of genes that show significant differential expression or splicing between worker honeybees and drones and between worker honeybees and queens. S15 Table: Molecular Function Gene Ontology (GO) of genes that show significant differential expression or splicing between worker honeybees and drones and between worker honeybees and queens. S1 Supporting Information: List of genes that are co-regulated by the sexual and caste signals.
(DOCX)
