# Supplementary material for: Linking Genes and Brain Development of Honeybee Workers: A Whole-Transcriptome Approach
Source: PLoS One. 2016 Aug 4;11(8):e0157980. doi: 10.1371/journal.pone.0157980 (PMC4973980; doi:10.1371/journal.pone.0157980)
Supplement: S1 File — S1 Figure: Venn diagram of the number of differentially expressed (DEGs) and differentially spliced (DSGs) genes in the worker/male and worker/queen comparisons. S1 Table: Numbers of reads that were sequenced and the numbers and proportions of reads that were mapped to the honeybee genome. S2 Table: Number of differentially expressed genes in the brain between the conditions. S3 Table: Number of differentially spliced genes (DSGs) between the conditions. S4 Table: Correlation coefficient of the FPKM values between the conditions and replicates. S5 Table: The average correlation coefficient of the FPKM values between the different conditions. S6 Table: Cellular Component Gene Ontology (GO) of differentially expressed genes between the pupal brains of worker bees and drones. S7 Table: Molecular Function Gene Ontology (GO) of differentially expressed genes between the pupal brains of worker bees and drones. S8 Table: Cellular Component Gene Ontology (GO) of genes that show significant differential use of splice junctions between the pupal brains of worker bees and drones. S9 Table: Molecular Function Gene Ontology (GO) of genes that show significant differential use of splice junctions between the pupal brains of worker bees and drones. S10 Table: Cellular Component Gene Ontology (GO) of significant differentially expressed genes between the pupal brains of worker bees and queens. S11 Table: Molecular Function Gene Ontology (GO) of significant differentially expressed genes between the pupal brains of worker bees and queens. S12 Table: Cellular Component Gene Ontology (GO) of genes that show significant differential use of splice junctions between the pupal brains of worker bees and queens. S13 Table: Molecular Function Gene Ontology (GO) of genes that show significant differential use of splice junctions between the pupal brains of worker bees and queens. S14 Table: Cellular Component Gene Ontology (GO) of genes that show significant differential expression or splicin [file pone.0157980.s001.docx]

# Supporting Information

**
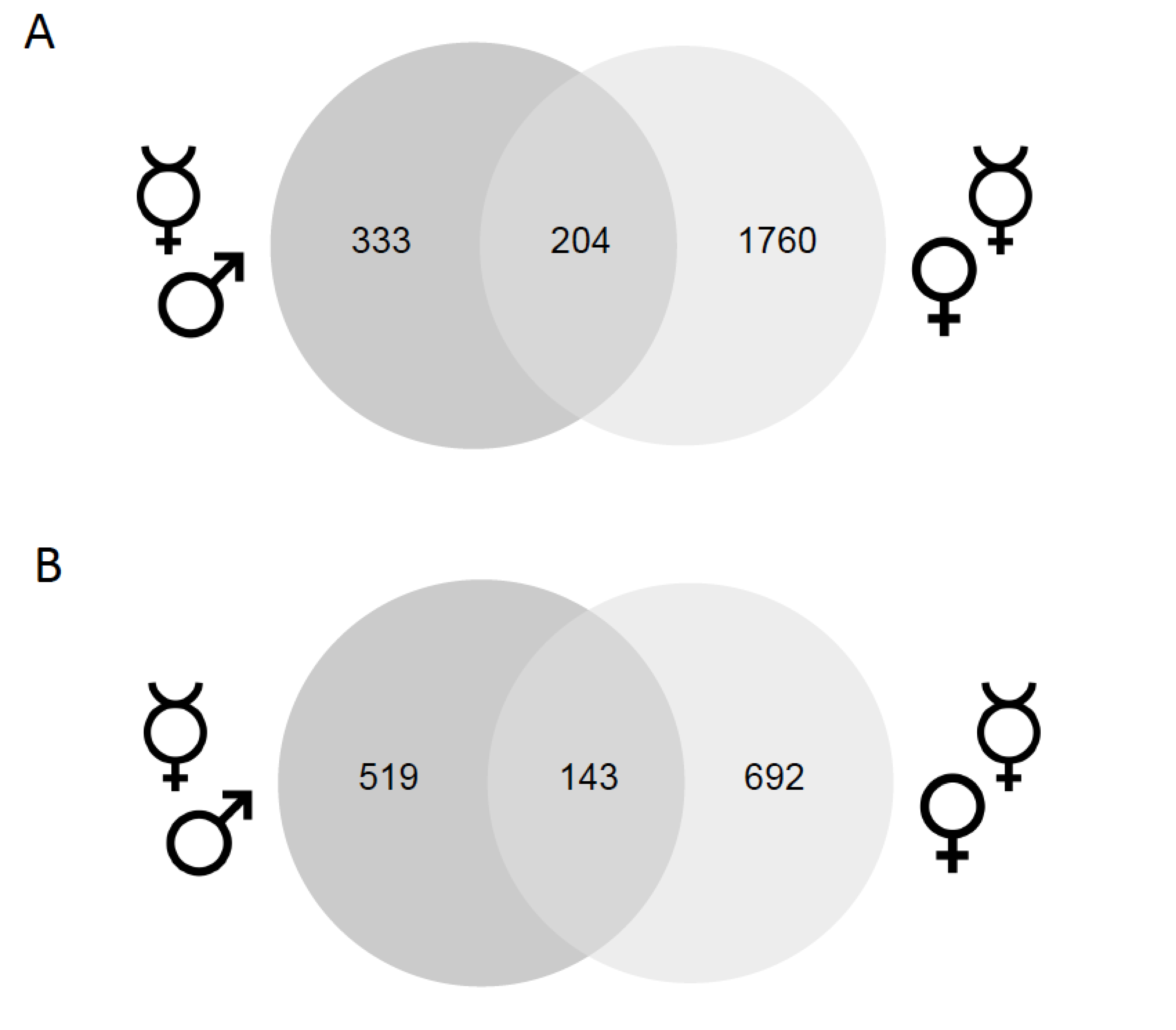
**

**S1 Figure: Venn diagram of the number of differently spliced and expressed genes in the worker/male and worker/queen contrasts. (A) DEGs and (B) DSGs are presented.**

**S1 Table: Numbers of reads that were sequenced and numbers and proportions of the reads that were mapped to the honeybee genome.** Conditions: W = Workers, D = Drones, Q = Queens; 1-3: replicates of each condition.

| **Condition and replicate** | **Number of**  **sequenced reads** | **Number of**  **mapped reads** | **Proportion of**  **mapped reads** |
| --- | --- | --- | --- |
| W1 | 175796462 | 141219986 | 80.33 % |
| W2 | 168267063 | 135826457 | 80.72 % |
| W3 | 178934269 | 143363696 | 80.12 % |
| D1 | 189487841 | 153435967 | 80.97 % |
| D2 | 162754857 | 131930626 | 81.06 % |
| D3 | 153692102 | 122870817 | 79.95 % |
| Q1 | 218188846 | 175755845 | 80.55 % |
| Q2 | 220797918 | 182387889 | 82.60 % |
| Q3 | 215385931 | 179337226 | 83.26 % |
| Mean | 187033921 | 151792057 | 81.06 % |

**S2 Table: Number of differential expressed genes in the brain between the conditions.** Differential expressed genes (*p* < 0.001) were estimated using the Cuffdiff program and are based on a *de novo* model of transcripts generated by the program Cufflinks.

|  | **Workers** | **Drones** | **Queens** |
| --- | --- | --- | --- |
| **Workers** | - | 333 | 1760 |
| **Drones** |  | - | 1763 |
| **Queens** |  |  | - |

**S3 Table: Number of differential spliced genes (DSGs) between the conditions.** Differential spliced junctions were estimated with the Spanki-junc command. Number in braces indicates the number of junctions, which are distributed over the number of DSGs.

|  | differentially spliced genes  (differentially spliced junctions) | | |
| --- | --- | --- | --- |
|  |  |  |  |
|  | **Workers** | **Drones** | **Queens** |
| **Workers** | - | 519 (817) | 627 (1150) |
| **Drones** |  | - | 811 (1408) |
| **Queen** |  |  | - |

**S4 Table: Correlation coefficient of the FPKM values between the conditions and replicates.** The FPKM values of the genes were compared using a Pearson's product-moment correlation. Conditions: W = Workers, D = Drones, Q = Queens; 1-3: replicates of each condition.

|  | **D1** | **D2** | **D3** | **W1** | **W2** | **W3** | **Q1** | **Q2** | **Q3** |
| --- | --- | --- | --- | --- | --- | --- | --- | --- | --- |
| **D1** | 1.00 | 0.95 | 0.97 | 0.87 | 0.91 | 0.89 | 0.69 | 0.68 | 0.67 |
| **D2** |  | 1.00 | 0.95 | 0.84 | 0.90 | 0.84 | 0.79 | 0.78 | 0.77 |
| **D3** |  |  | 1.00 | 0.90 | 0.94 | 0.91 | 0.70 | 0.69 | 0.68 |
| **W1** |  |  |  | 1.00 | 0.95 | 0.96 | 0.60 | 0.58 | 0.58 |
| **W2** |  |  |  |  | 1.00 | 0.90 | 0.67 | 0.65 | 0.64 |
| **W3** |  |  |  |  |  | 1.00 | 0.63 | 0.61 | 0.61 |
| **Q1** |  |  |  |  |  |  | 1.00 | 0.99 | 0.99 |
| **Q2** |  |  |  |  |  |  |  | 1.00 | 0.99 |
| **Q3** |  |  |  |  |  |  |  |  | 1.00 |

**S5 Table: The average correlation coefficient of the FPKM values between the different conditions (obtained from S4 Table).**

|  | **Workers** | **Drones** | **Queens** |
| --- | --- | --- | --- |
| **Workers** | 0.93 | 0.89 | 0.61 |
| **Drones** |  | 0.95 | 0.71 |
| **Queens** |  |  | 0.99 |

**S6 Table: Cellular Component Gene ontology (GO) of differential expressed genes between the pupal brains of worker bees and drones.** GO terms significantly enriched (p<0.01, Fisher’s exact test) in the gene list are presented. GO terms were derived by DAVID Bioinformatic Resources and summarized by REVIGO software. The frequency (>0.01%) of the genes in the Cellular Component group and the p-value is listed.

| **Gene ontology term ID** | **Description** | **Frequency** | **p-value** |
| --- | --- | --- | --- |
| GO:0005576 | extracellular region | 4.57% | 0.0079 |
| GO:0005783 | endoplasmic reticulum | 0.30% | 0.0289 |
| GO:0000267 | cell fraction | 0.16% | 0.0023 |
| GO:0005624 | membrane fraction | 0.16% | 0.0017 |
| GO:0005626 | insoluble fraction | 0.16% | 0.0020 |
| GO:0015629 | actin cytoskeleton | 0.15% | 0.0354 |
| GO:0019898 | extrinsic component of membrane | 0.10% | 0.0016 |
| GO:0030016 | myofibril | 0.03% | 0.0076 |
| GO:0030017 | sarcomere | 0.03% | 0.0067 |
| GO:0005861 | troponin complex | 0.01% | 0.0166 |

**S7 Table: Molecular Function Gene ontology (GO) of differential expressed genes between the pupal brains of worker bees and drones.** GO terms significantly enriched (p<0.01, Fisher’s exact test) in the gene list are presented. GO terms were derived by DAVID Bioinformatic Resources and summarized by REVIGO software. The frequency (>0.01%) of the genes in the Molecular Function group and the p-value is listed.

| **Gene ontology term ID** | **Description** | **Frequency** | **p-value** |
| --- | --- | --- | --- |
| GO:0003700 | sequence-specific DNA binding transcription factor activity | 4.96% | 0.0048 |
| GO:0043565 | sequence-specific DNA binding | 1.94% | 0.0106 |
| GO:0046906 | tetrapyrrole binding | 1.91% | 0.0368 |
| GO:0020037 | heme binding | 1.77% | 0.0368 |
| GO:0030170 | pyridoxal phosphate binding | 1.70% | 0.0143 |
| GO:0008236 | serine-type peptidase activity | 1.04% | 0.0491 |
| GO:0016769 | transferase activity, transferring nitrogenous groups | 0.82% | 0.0305 |
| GO:0004252 | serine-type endopeptidase activity | 0.68% | 0.0306 |
| GO:0015293 | symporter activity | 0.58% | 0.0243 |
| GO:0019842 | vitamin binding | 0.56% | 0.0019 |
| GO:0015294 | solute:cation symporter activity | 0.49% | 0.0207 |
| GO:0016782 | transferase activity, transferring sulfur-containing groups | 0.49% | 0.0485 |
| GO:0015370 | solute:sodium symporter activity | 0.36% | 0.0477 |
| GO:0030528 | transcription regulator activity | 0.16% | 0.0322 |
| GO:0008146 | sulfotransferase activity | 0.03% | 0.0182 |
| GO:0017051 | retinol dehydratase activity | 0.01% | 0.0261 |

**S8 Table: Cellular Component Gene ontology (GO) of genes with significant differential used splice junctions between the pupal brains of worker bees and drones.** GO terms significantly enriched (p<0.01, Fisher’s exact test) in the gene list are presented. GO terms were derived by DAVID Bioinformatic Resources and summarized by REVIGO software. The frequency (>0.01%) of the genes in the Cellular Component group and the p-value is listed.

| **Gene ontology term ID** | **Description** | **Frequency** | **p-value** | |
| --- | --- | --- | --- | --- |
| GO:0031224 | intrinsic component of membrane | 23.87% | 0.0410 | |
| GO:0016021 | integral component of membrane | 23.61% | 0.0328 | |
| GO:0005856 | cytoskeleton | 7.66% | 0.0378 | |
| GO:0005887 | integral component of plasma membrane | 3.23% | 0.0890 | |
| GO:0045202 | synapse | 3.05% | 0.0338 | |
| GO:0030054 | cell junction | 2.49% | 0.0011 | |
| GO:0043005 | neuron projection | 2.39% | 0.0100 | |
| GO:0005938 | cell cortex | 1.64% | 0.0147 | |
| GO:0015629 | actin cytoskeleton | 1.53% | 0.0263 | |
| GO:0005700 | polytene chromosome | 1.49% | 0.0325 | |
| GO:0044449 | contractile fiber part | 1.07% | 0.0467 | |
| GO:0005911 | cell-cell junction | 1.00% | 0.0043 | |
| GO:0044448 | cell cortex part | 0.92% | 0.0117 | |
| GO:0034703 | cation channel complex | 0.80% | 0.0164 | |
| GO:0043296 | apical junction complex | 0.70% | 0.0078 | |
| GO:0016324 | apical plasma membrane | 0.55% | 0.0095 | |
| GO:0070160 | occluding junction | 0.45% | 0.0134 | |
| GO:0005918 | septate junction | 0.44% | 0.0073 | |
| GO:0000139 | Golgi membrane | 0.39% | 0.0445 | |
| GO:0016327 | apicolateral plasma membrane | 0.22% | 0.0108 | |
|  | | | |  |

**S9 Table: Molecular Function Gene ontology (GO) of genes with significant differential used splice junctions between the pupal brains of worker bees and drones.** GO terms significantly enriched (p<0.01, Fisher’s exact test) in the gene list are presented. GO terms were derived by DAVID Bioinformatic Resources and summarized by REVIGO software. The frequency (>0.01%) of the genes in the Molecular Function group and the p-value is listed.

| **Gene ontology term ID** | **Description** | **Frequency** | **p-value** |
| --- | --- | --- | --- |
| GO:0017076 | purine nucleotide binding | 11.88% | 0.0322 |
| GO:0001882 | nucleoside binding | 11.78% | 0.0322 |
| GO:0001883 | purine nucleoside binding | 11.74% | 0.0490 |
| GO:0030554 | adenyl nucleotide binding | 10.31% | 0.0462 |
| GO:0004672 | protein kinase activity | 4.60% | 0.0328 |
| GO:0008092 | cytoskeletal protein binding | 3.69% | 0.0055 |
| GO:0005509 | calcium ion binding | 2.28% | 0.0044 |
| GO:0022836 | gated channel activity | 2.04% | 0.0331 |
| GO:0004713 | protein tyrosine kinase activity | 1.49% | 0.0411 |
| GO:0008509 | anion transmembrane transporter activity | 0.93% | 0.0418 |
| GO:0015293 | symporter activity | 0.42% | 0.0077 |
| GO:0015294 | solute:cation symporter activity | 0.39% | 0.0061 |
| GO:0019205 | nucleobase-containing compound kinase activity | 0.39% | 0.0196 |
| GO:0015103 | inorganic anion transmembrane transporter activity | 0.38% | 0.0434 |
| GO:0015370 | solute:sodium symporter activity | 0.33% | 0.0090 |
| GO:0019201 | nucleotide kinase activity | 0.27% | 0.0423 |
| GO:0015296 | anion:cation symporter activity | 0.10% | 0.0406 |

**S10 Table: Cellular Component Gene ontology (GO) of significant differential expressed genes between the pupal brains of worker bees and queens.** GO terms significantly enriched (p<0.01, Fisher’s exact test) in the gene list are presented. GO terms were derived by DAVID Bioinformatic Resources and summarized by REVIGO software. The frequency (>0.01%) of the genes in the Cellular Component group and the p-value is listed.

| **Gene ontology term ID** | **Description** | | **Frequency** | | **p-value** | |
| --- | --- | --- | --- | --- | --- | --- |
| GO:0005886 | | plasma membrane | | 12.71% | 0.0000 | |
| GO:0005576 | | extracellular region | | 8.65% | 0.0189 | |
| GO:0005856 | | cytoskeleton | | 7.66% | 0.0058 | |
| GO:0044430 | | cytoskeletal part | | 6.79% | 0.0021 | |
| GO:0044459 | | plasma membrane part | | 5.56% | 0.0000 | |
| GO:0044421 | | extracellular region part | | 4.31% | 0.0051 | |
| GO:0005875 | | microtubule associated complex | | 3.66% | 0.0164 | |
| GO:0031226 | | intrinsic component of plasma membrane | | 3.35% | 0.0498 | |
| GO:0005887 | | integral component of plasma membrane | | 3.23% | 0.0435 | |
| GO:0030054 | | cell junction | | 2.49% | 0.0012 | |
| GO:0005811 | | lipid particle | | 1.96% | 0.0057 | |
| GO:0005938 | | cell cortex | | 1.64% | 0.0400 | |
| GO:0015629 | | actin cytoskeleton | | 1.53% | 0.0001 | |
| GO:0070161 | | anchoring junction | | 1.11% | 0.0018 | |
| GO:0005912 | | adherens junction | | 1.11% | 0.0015 | |
| GO:0043292 | | contractile fiber | | 1.08% | 0.0000 | |
| GO:0030016 | | myofibril | | 1.07% | 0.0041 | |
| GO:0044449 | | contractile fiber part | | 1.07% | 0.0001 | |
| GO:0030017 | | sarcomere | | 1.05% | 0.0030 | |
| GO:0005911 | | cell-cell junction | | 1.00% | 0.0169 | |
| GO:0005667 | | transcription factor complex | | 0.87% | 0.0262 | |
| GO:0005871 | | kinesin complex | | 0.66% | 0.0177 | |
| GO:0045169 | | fusome | | 0.56% | 0.0055 | |
| GO:0016459 | | myosin complex | | 0.49% | 0.0018 | |
| GO:0019898 | | extrinsic component of membrane | | 0.45% | 0.0013 | |
| GO:0044445 | | cytosolic part | | 0.39% | 0.0367 | |
| GO:0045171 | | intercellular bridge | | 0.32% | 0.0041 | |
| GO:0045172 | | germline ring canal | | 0.30% | 0.0041 | |
| GO:0019897 | | extrinsic component of plasma membrane | | 0.26% | 0.0033 | |
| GO:0031225 | | anchored component of membrane | | 0.17% | 0.0355 | |
| GO:0005865 | | striated muscle thin filament | | 0.13% | 0.0443 | |
| GO:0016460 | | myosin II complex | | 0.03% | 0.0103 | |
| GO:0005832 | | chaperonin-containing T-complex | | 0.02% | 0.0156 | |
| GO:0022625 | | cytosolic large ribosomal subunit | | 0.02% | 0.0151 | |
| GO:0005859 | | muscle myosin complex | | 0.02% | 0.0033 | |
| GO:0019908 | | nuclear cyclin-dependent protein kinase holoenzyme complex | | 0.01% | 0.0443 | |
|  | | | | | |  |

**S11 Table: Molecular Function Gene ontology (GO) of significant differential expressed genes between the pupal brains of worker bees and queens.** GO terms significantly enriched (p<0.01, Fisher’s exact test) in the gene list are presented. GO terms were derived by DAVID Bioinformatic Resources and summarized by REVIGO software. The frequency (>0.01%) of the genes in the Molecular Function group and the p-value is listed.

| **Gene ontology term ID** | **Description** | | **Frequency** | | **p-value** | |
| --- | --- | --- | --- | --- | --- | --- |
| GO:0043167 | | ion binding | | 29.29% | 0.0045 |  |
| GO:0000166 | | nucleotide binding | | 15.15% | 0.0132 |  |
| GO:0017076 | | purine nucleotide binding | | 11.88% | 0.0115 |  |
| GO:0032553 | | ribonucleotide binding | | 11.87% | 0.0221 |  |
| GO:0001882 | | nucleoside binding | | 11.78% | 0.0115 |  |
| GO:0032555 | | purine ribonucleotide binding | | 11.76% | 0.0176 |  |
| GO:0001883 | | purine nucleoside binding | | 11.74% | 0.0221 |  |
| GO:0030554 | | adenyl nucleotide binding | | 10.31% | 0.0214 |  |
| GO:0032559 | | adenyl ribonucleotide binding | | 10.20% | 0.0205 |  |
| GO:0005524 | | ATP binding | | 10.18% | 0.0001 |  |
| GO:0003677 | | DNA binding | | 7.96% | 0.0003 |  |
| GO:0008092 | | cytoskeletal protein binding | | 3.69% | 0.0000 |  |
| GO:0003700 | | sequence-specific DNA binding transcription factor activity | | 3.25% | 0.0000 |  |
| GO:0043565 | | sequence-specific DNA binding | | 3.17% | 0.0115 |  |
| GO:0005509 | | calcium ion binding | | 2.28% | 0.0001 |  |
| GO:0003779 | | actin binding | | 1.81% | 0.0267 |  |
| GO:0015631 | | tubulin binding | | 1.36% | 0.0163 |  |
| GO:0008017 | | microtubule binding | | 1.27% | 0.0001 |  |
| GO:0003774 | | motor activity | | 1.06% | 0.0072 |  |
| GO:0003777 | | microtubule motor activity | | 0.70% | 0.0462 |  |
| GO:0016566 | | specific transcriptional repressor activity | | 0.64% | 0.0061 |  |
| GO:0016564 | | transcription repressor activity | | 0.64% | 0.0000 |  |
| GO:0003702 | | RNA polymerase II transcription factor activity | | 0.64% | 0.0000 |  |
| GO:0003704 | | specific RNA polymerase II transcription factor activity | | 0.64% | 0.0000 |  |
| GO:0030528 | | transcription regulator activity | | 0.64% | 0.0068 |  |
| GO:0051015 | | actin filament binding | | 0.38% | 0.0372 |  |
| GO:0015370 | | solute:sodium symporter activity | | 0.33% | 0.0105 |  |
| GO:0003707 | | steroid hormone receptor activity | | 0.22% | 0.0010 |  |
| GO:0004879 | | ligand-activated sequence-specific DNA binding RNA polymerase II transcription factor activity | | 0.15% | 0.0090 |  |
| GO:0004715 | | non-membrane spanning protein tyrosine kinase activity | | 0.11% | 0.0015 |  |
| GO:0000146 | | microfilament motor activity | | 0.07% | 0.0009 |  |
| GO:0015929 | | hexosaminidase activity | | 0.05% | 0.0045 |  |

**S12 Table: Cellular Component Gene ontology (GO) of genes with significant differential used splice junctions between the pupal brains of worker bees and queens.** GO terms significantly enriched (p<0.01, Fisher’s exact test) in the gene list are presented. GO terms were derived by DAVID Bioinformatic Resources and summarized by REVIGO software. The frequency (>0.01%) of the genes in the Cellular Component group and the p-value is listed.

| **Gene ontology term ID** | **Description** | **Frequency** | **p-value** |  |
| --- | --- | --- | --- | --- |
| GO:0044421 | extracellular region part | 4.31% | 0.0428 | |
| GO:0042995 | cell projection | 3.37% | 0.0081 | |
| GO:0043005 | neuron projection | 2.39% | 0.0269 | |
| GO:0005811 | lipid particle | 1.96% | 0.0051 | |
| GO:0031012 | extracellular matrix | 1.70% | 0.0486 | |
| GO:0030424 | axon | 1.66% | 0.0292 | |
| GO:0005874 | microtubule | 1.54% | 0.0386 | |
| GO:0015629 | actin cytoskeleton | 1.53% | 0.0018 | |
| GO:0044463 | cell projection part | 1.36% | 0.0356 | |
| GO:0005912 | adherens junction | 1.11% | 0.0333 | |
| GO:0070161 | anchoring junction | 1.11% | 0.0356 | |
| GO:0045211 | postsynaptic membrane | 0.79% | 0.0290 | |
| GO:0043296 | apical junction complex | 0.70% | 0.0213 | |
| GO:0005578 | proteinaceous extracellular matrix | 0.54% | 0.0406 | |
| GO:0070160 | occluding junction | 0.45% | 0.0292 | |
| GO:0005925 | focal adhesion | 0.25% | 0.0437 | |
| GO:0044420 | extracellular matrix part | 0.24% | 0.0437 | |
| GO:0016327 | apicolateral plasma membrane | 0.22% | 0.0059 | |
| GO:0030863 | cortical cytoskeleton | 0.21% | 0.0095 | |
| GO:0005604 | basement membrane | 0.21% | 0.0376 | |
| GO:0009898 | cytoplasmic side of plasma membrane | 0.06% | 0.0269 | |

**S13 Table: Molecular Function Gene ontology (GO) of genes with significant differential used splice junctions between the pupal brains of worker bees and queens.** GO terms significantly enriched (p<0.01, Fisher’s exact test) in the gene list are presented. GO terms were derived by DAVID Bioinformatic Resources and summarized by REVIGO software. The frequency (>0.01%) of the genes in the Molecular Function group and the p-value is listed.

| **Gene ontology term ID** | **Description** | **Frequency** | **p-value** |
| --- | --- | --- | --- |
| GO:0043167 | ion binding | 29.29% | 0.0241 |
| GO:0043169 | cation binding | 17.07% | 0.0433 |
| GO:0046872 | metal ion binding | 16.80% | 0.0369 |
| GO:0000166 | nucleotide binding | 15.15% | 0.0044 |
| GO:0032553 | ribonucleotide binding | 11.87% | 0.0025 |
| GO:0032555 | purine ribonucleotide binding | 11.76% | 0.0025 |
| GO:0032559 | adenyl ribonucleotide binding | 10.20% | 0.0035 |
| GO:0005524 | ATP binding | 10.18% | 0.0034 |
| GO:0016887 | ATPase activity | 4.10% | 0.0107 |
| GO:0022890 | inorganic cation transmembrane transporter activity | 3.27% | 0.0311 |
| GO:0042623 | ATPase activity, coupled | 2.93% | 0.0120 |
| GO:0070279 | vitamin B6 binding | 2.50% | 0.0295 |
| GO:0046873 | metal ion transmembrane transporter activity | 2.46% | 0.0384 |
| GO:0048037 | cofactor binding | 1.68% | 0.0014 |
| GO:0050662 | coenzyme binding | 1.29% | 0.0252 |
| GO:0008289 | lipid binding | 0.88% | 0.0303 |
| GO:0004714 | transmembrane receptor protein tyrosine kinase activity | 0.86% | 0.0046 |
| GO:0051015 | actin filament binding | 0.38% | 0.0127 |
| GO:0030170 | pyridoxal phosphate binding | 0.34% | 0.0295 |
| GO:0051119 | sugar transmembrane transporter activity | 0.04% | 0.0083 |

**S14 Table: Cellular Component Gene ontology (GO) of genes that are significant differential expressed or spliced between both worker honeybees and drones and between worker honeybees and queens.** GO terms significantly enriched (p<0.01, Fisher’s exact test) in the gene list are presented. GO terms were derived by DAVID Bioinformatic Resources and summarized by REVIGO software. The frequency (>0.01%) of the genes in the Cellular Component group and the p-value is listed.

| **Gene ontology term ID** | **Description** | **Frequency** | **p-value** |
| --- | --- | --- | --- |
| GO:0005886 | plasma membrane | 12.08% | 0.0012 |
| GO:0005576 | extracellular region | 9.54% | 0.0155 |
| GO:0044421 | extracellular region part | 4.67% | 0.0328 |
| GO:0044459 | plasma membrane part | 4.67% | 0.0197 |
| GO:0015629 | actin cytoskeleton | 1.54% | 0.0035 |
| GO:0019898 | extrinsic to membrane | 0.42% | 0.0094 |
| GO:0016323 | basolateral plasma membrane | 0.40% | 0.0377 |
| GO:0005865 | striated muscle thin filament | 0.06% | 0.0014 |
| GO:0005861 | troponin complex | 0.06% | 0.0237 |

**S15 Table: Molecular Function Gene ontology (GO) of genes that are significant differential expressed or spliced between both worker honeybees and drones and between worker honeybees and queens.** GO terms significantly enriched (p<0.01, Fisher’s exact test) in the gene list are presented. GO terms were derived by DAVID Bioinformatic Resources and summarized by REVIGO software. The frequency (>0.01%) of the genes in the Molecular Function group and the p-value is listed.

| **Gene ontology term ID** | **Description** | **Frequency** | **p-value** |
| --- | --- | --- | --- |
| GO:0005509 | calcium ion binding | 2.06% | 0.0017 |
| GO:0001633 | secretin-like receptor activity | 0.66% | 0.0197 |

**S1 Supporting Information: List of genes that are co-regulated by the sexual and caste signal.**

| Aats-tyr | LOC100576877 | LOC408394 | LOC411298 | LOC552523 | LOC727129 |
| --- | --- | --- | --- | --- | --- |
| Abd-A | LOC100576895 | LOC408396 | LOC411317 | LOC552543 | LOC727131 |
| Aldh | LOC100576899 | LOC408463 | LOC411330 | LOC552636 | LOC727136 |
| Aplip1 | LOC100576916 | LOC408522 | LOC411347 | LOC552685 | LOC727232 |
| Arr3 | LOC100576929 | LOC408565 | LOC411387 | LOC552738 | LOC727284 |
| blow | LOC100576949 | LOC408650 | LOC411463 | LOC552799 | LOC727293 |
| BM-40-SPARC | LOC100577004 | LOC408696 | LOC411564 | LOC724156 | LOC727649 |
| btsz | LOC100577045 | LOC408699 | LOC411609 | LOC724172 | MAPKKK9 |
| CalpC | LOC100577045 | LOC408718 | LOC411665 | LOC724195 | Mf |
| chp | LOC100577064 | LOC408857 | LOC411692 | LOC724208 | Mhc1 |
| Cht3 | LOC100577149 | LOC408864 | LOC411748 | LOC724239 | Mical |
| Cht5 | LOC100577165 | LOC408868 | LOC411905 | LOC724286 | mio |
| Clc | LOC100577229 | LOC408871 | LOC411978 | LOC724292 | Mlc2 |
| CPF2 | LOC100577390 | LOC408917 | LOC412020 | LOC724440 | Mrjp7 |
| CPR15 | LOC100577410 | LOC409057 | LOC412030 | LOC724460 | mRpS29 |
| crc | LOC100577440 | LOC409060 | LOC412157 | LOC724518 | mwh |
| CTL12 | LOC100577478 | LOC409071 | LOC412162 | LOC724563 | Nep2 |
| CTL4 | LOC100577504 | LOC409163 | LOC412167 | LOC724693 | Nep5 |
| CYP305D1 | LOC100577576 | LOC409187 | LOC412588 | LOC724746 | NT-10 |
| CYP6AR1 | LOC100577586 | LOC409235 | LOC412784 | LOC724749 | Obp13 |
| Cyp6as5 | LOC100577625 | LOC409306 | LOC412842 | LOC724825 | Obp18 |
| CYP9Q1 | LOC100577646 | LOC409327 | LOC412885 | LOC724900 | Obp3 |
| CYP9Q2 | LOC100577680 | LOC409348 | LOC412918 | LOC724922 | Osi17 |
| Dat | LOC100577718 | LOC409425 | LOC412948 | LOC725041 | Osi18 |
| dbo | LOC100577832 | LOC409463 | LOC412996 | LOC725065 | Osi19 |
| Dfd | LOC100577839 | LOC409598 | LOC413005 | LOC725155 | Osi20 |
| Dh31-R1 | LOC100577843 | LOC409613 | LOC413021 | LOC725215 | Osi7 |
| DI | LOC100577845 | LOC409620 | LOC413043 | LOC725218 | Osi9 |
| Dop1 | LOC100577887 | LOC409655 | LOC413048 | LOC725259 | Pcd |
| DopR2 | LOC100577890 | LOC409665 | LOC413697 | LOC725294 | Pkcdelta |
| dyl | LOC100577912 | LOC409718 | LOC413785 | LOC725318 | PLA2-2.4 |
| ebony | LOC100577924 | LOC409752 | LOC413858 | LOC725361 | pot |
| fng | LOC100578085 | LOC409771 | LOC413936 | LOC725381 | PPO |
| fus | LOC100578094 | LOC409776 | LOC413942 | LOC725389 | pwn |
| Gmap | LOC100578137 | LOC409780 | LOC413976 | LOC725422 | Pxd |
| GMCOX1 | LOC100578193 | LOC409781 | LOC413987 | LOC725462 | Pyx2 |
| Grx-like1 | LOC100578226 | LOC409814 | LOC413994 | LOC725507 | RabGAP6 |
| GST-mic2 | LOC100578278 | LOC409950 | LOC414029 | LOC725634 | RhoBTB |
| GstD1 | LOC100578296 | LOC409970 | LOC550977 | LOC725697 | RhoGDI |
| Hex70a | LOC100578316 | LOC410001 | LOC551089 | LOC725699 | Roc1a |
| Hil | LOC100578345 | LOC410038 | LOC551137 | LOC725724 | Rx |
| hipk | LOC100578397 | LOC410057 | LOC551167 | LOC725729 | Scp2 |
| Ipp | LOC100578419 | LOC410087 | LOC551170 | LOC725733 | SCR-B5 |
| kinesin-2C | LOC100578457 | LOC410092 | LOC551171 | LOC725903 | SP10 |
| LOC100188904 | LOC100578464 | LOC410187 | LOC551291 | LOC725922 | SP16 |
| LOC100576090 | LOC100578540 | LOC410201 | LOC551364 | LOC725965 | SP25 |
| LOC100576121 | LOC100578572 | LOC410261 | LOC551437 | LOC725969 | SP33 |
| LOC100576127 | LOC100578606 | LOC410274 | LOC551474 | LOC726001 | SP5 |
| LOC100576157 | LOC100578615 | LOC410282 | LOC551493 | LOC726020 | SP6 |
| LOC100576192 | LOC100578623 | LOC410320 | LOC551496 | LOC726021 | SP8 |
| LOC100576214 | LOC100578625 | LOC410334 | LOC551553 | LOC726025 | SPH42 |
| LOC100576234 | LOC100578635 | LOC410417 | LOC551587 | LOC726045 | SPH51 |
| LOC100576258 | LOC100578665 | LOC410452 | LOC551613 | LOC726094 | spz2 |
| LOC100576271 | LOC100578672 | LOC410483 | LOC551650 | LOC726125 | Syt20 |
| LOC100576318 | LOC100578678 | LOC410532 | LOC551761 | LOC726201 | TpnCIIb |
| LOC100576418 | LOC100578685 | LOC410649 | LOC551897 | LOC726268 | TpnI |
| LOC100576454 | LOC100578698 | LOC410729 | LOC551911 | LOC726320 | TpnT |
| LOC100576473 | LOC100578727 | LOC410730 | LOC551920 | LOC726514 | TRPML |
| LOC100576488 | LOC100578732 | LOC410829 | LOC551973 | LOC726625 | Twdl2 |
| LOC100576497 | LOC100578744 | LOC410884 | LOC551994 | LOC726657 | Vhdl |
| LOC100576580 | LOC100578770 | LOC410975 | LOC552005 | LOC726672 | Wat |
| LOC100576603 | LOC100578822 | LOC411011 | LOC552158 | LOC726758 | Y-b |
| LOC100576659 | LOC100578899 | LOC411023 | LOC552261 | LOC726795 | Y-y |
| LOC100576773 | LOC100579047 | LOC411090 | LOC552311 | LOC726817 |  |
| LOC100576841 | LOC406114 | LOC411186 | LOC552356 | LOC726842 |  |
| LOC100578835 | LOC406147 | LOC411229 | LOC552372 | LOC726864 |  |
| LOC100578860 | LOC408299 | LOC411272 | LOC552421 | LOC726990 |  |
| LOC100578866 | LOC408318 | LOC411285 | LOC552446 | LOC727007 |  |
